# A mixed methods study to examine the influence of the neighborhood social context on adolescent health service utilization

**DOI:** 10.1186/s12913-016-1597-x

**Published:** 2016-08-24

**Authors:** Kristin Mmari, Beth Marshall, Trevor Hsu, Ji Won Shon, Amenze Eguavoen

**Affiliations:** 1 Johns Hopkins Bloomberg School of Public Health, 615 N. Wolfe Street, Baltimore, MD 21205 USA; 2 Rollins School of Public Health, Emory University, 1518 Clifton Road, Atlanta, GA 30322 USA; 3 Pathfinder International, 38b Moshood Abiola Crescent, Off Toyin Street, Ikeja, Lagos State Nigeria

**Keywords:** Adolescents, Health seeking behaviors, Cross-cultural research, Social factors

## Abstract

**Background:**

While adolescents’ access and utilization of health services is critical for ensuring their health, very few seek care, and if they do, it is primarily from family members, friends, or other non-formal sources of care. Examining the influence of the social context on adolescent health care seeking behaviors may provide us with a better understanding for how interventions can increase adolescents’ utilization of formal health care services.

**Methods:**

The study is based on qualitative and quantitative data collected as part of the Well Being of Adolescents in Vulnerable Environments (WAVE) study, one of the first global studies to focus on very disadvantaged urban adolescents (aged 15–19 years) across five diverse sites, which include: Baltimore (USA), Ibadan (Nigeria), Johannesburg (South Africa), New Delhi (India), and Shanghai (China). Qualitative data was based on numerous methodologies, including key informant interviews, a Photovoice exercise, community mapping, focus groups and in-depth interviews. Quantitative data was gathered from a cross-sectional Audio Computer Assisted Self Interview (ACASI) survey that was administered to approximately 450–500 adolescents per site, yielding a total of 2,393 adolescents. Respondent-driven sampling was used to ensure the sample include out-of-school youth and unstably housed youth who are often underrepresented in school-based or household-based samples.

**Results:**

While adolescents in Baltimore, New Delhi, and Johannesburg were more likely to seek health services if they felt illness symptoms, a fairly large proportion of adolescents indicated that even when they needed health care, they didn’t seek it. In Johannesburg, more than 30 % of adolescents did not seek care even when they knew it was needed. Similarly, nearly a quarter of adolescents in Baltimore and in Shanghai indicated not seeking care when needed. Qualitative data indicated that adolescents exhibited a general lack of trust in providers and often felt embarrassed or stigmatized for seeking services. Multivariate analysis revealed that perceived fear and exposure to community violence was associated with a decreased likelihood of seeking care, while adult support from the home increased adolescents’ likelihood to seek care in Baltimore and Johannesburg.

**Conclusions:**

Adolescent health care seeking patterns vary substantially by setting and gender. Neighborhood and family environments are important contexts in which health seeking behaviors are shaped. Efforts to connect adolescents to health care will need to target neighborhood safety as well as trust and support among adults outside of provider settings.

**Electronic supplementary material:**

The online version of this article (doi:10.1186/s12913-016-1597-x) contains supplementary material, which is available to authorized users.

## Background

Despite the prevailing notion that adolescents are in one of the healthiest life stages, adolescents of today face more challenges to their health and development than they did a generation ago [1]. Many of these challenges can be attributed to the effects of globalization, technological advances, and economic development. Yet, poor adolescents living in slums and inner city environments bear the brunt of these health challenges, especially those in Sub-Saharan Africa and South Asia, where both poverty and population rates have been rising steadily [2]. An analysis of data from demographic and health surveys, for example, showed that the poorest 20 % of young women are between 1.7 and 4.0 times more likely to have an early birth compared to the richest 20 % of young women [3]. In fact, irrespective of whether it is a high-income or low-income country, poor urban adolescents face increased risks to a variety of health problems, including sexually transmitted infections [4, 5], substance abuse [6, 7] and road traffic injuries [8, 9]. Given that most of these health problems are largely preventable, utilization of preventative and curative health services is viewed as a critical component for improving adolescent health and well-being [1]. Yet research has shown that adolescents do not seek health care from formal health facilities, and instead, primarily rely on friends and family members to meet their health needs [10, 11].

With the growth of adolescents and young adults living in poor urban environments only expected to increase, there is indeed cause for concern that health challenges facing this population will also continue to increase. While decades of research has described numerous barriers facing adolescents when they do seek health services, even when health service initiatives (often referred to as ‘adolescent friendly’ or ‘youth friendly’) attempt to address these barriers, the evidence for such initiatives have been mixed [12–15].

To understand more clearly the reasons why adolescents under-utilize health services, it may be worthwhile to examine the social context in which health care seeking behaviors occur. After all, since the majority of adolescents meet their health care needs by their friends and family [16, 17], understanding how adolescents’ social contexts influence health seeking may provide us with a better idea for how ‘youth friendly’ initiatives can improve service utilization.

Key aspects within the social context that may be especially relevant to health care seeking behaviors are adolescents’ social support structures and networks, which are most commonly formed within neighborhoods, families, and peers at schools [18]. Previous research among adults has shown that within a neighborhood, the availability of social structures such as recreation centers, community gardens, and parks provide people with a “feel good” attitude towards their environment and enables social interaction [18]. In disadvantaged neighborhoods, often times these types of structures are either non-existent or in poor shape, with violence and criminal activities preventing residents from interacting with each other – leading to a lower sense of social support at the neighborhood level [19]. Even in neighborhoods characterized as ‘poor’ and ‘dirty’, however, there is some evidence that if residents feel safe and have a shared sense of values, they make use of local facilities which contribute to maintaining their health. On the other hand, having a sense of fear (or lack of safety) creates an environment that undermines mental and physical health, and consequently, research has shown that adult residents don’t seek help or services [20–22]. Whether this is true among adolescents, however, remains to be seen.

At the family level, while many families can serve as a ‘bridge’ to health care, they can also be barriers to receiving services [16, 17]. In some settings, adolescents may attempt to spare their parents from information that they know will make them uncomfortable, such as reproductive health information, and as a result, may turn to friends or other family members for health care. Whether this may be a function of how often parents and adolescents communicate with each other, or the extent to which the parent-child relationship is characterized as supportive, is not yet known, but since parents can facilitate or hinder an adolescents’ access to health services, examining the level of parental support may be worthwhile. Additionally, while it is widely known that peer support for certain health behaviors is important for adolescents [23], it is not yet known whether perceived peer support can also influence health care seeking behaviors.

To examine the influence of family, peer, and neighborhood social supports in relation to health care seeking among adolescents living in disadvantaged urban environments, this study utilized qualitative and quantitative data collected as part of the Well Being of Adolescents in Vulnerable Environments (WAVE) study. The WAVE study is actually one of the first global studies to focus on very disadvantaged urban adolescents (aged 15-19 years) across five diverse sites, which include: Baltimore (USA), Ibadan (Nigeria), Johannesburg (South Africa), New Delhi (India), and Shanghai (China). For further details about each of these study sites, please refer to Additional file 1. Using this particular study for our analysis provides an excellent opportunity to examine not only how adolescent health care seeking behaviors differ across urban sites, but also to understand the variations in the way social factors influence adolescent health care seeking behaviors across such sites.

## Methods

### Research design

The first phase of the study, the qualitative and formative phase, was launched in June of 2011 to: 1) explore adolescents’ perceived health and their top health challenges and; 2) describe the factors within their urban communities which were perceived to be related to their health and health seeking behaviors. Data were collected using identical research protocols across the five study sites: key informant interviews among representatives from schools, places of worship, and youth-serving organizations; in-depth interviews among adolescents; community mapping and focus groups among adolescents; and a Photovoice exercise among adolescents (for further details of the methodology of this phase, refer to [24]).

For the second phase of WAVE, a survey using Audio Computer Assisted Self Interview (ACASI) technology was conducted in the spring and summer of 2013 among adolescents in the same five urban sites [25]. Prior to programming the ACASI, the survey instrument was translated by an in-country native speaker and then back translated by a native speaker studying in the US. Both translators worked together to address any discrepancies. A native speaker then recorded the audio for the translated survey. The purpose of the survey was to examine the major health issues and perceived risk factors identified in the qualitative phase more broadly across study sites. To recruit adolescents for the survey, each site used a respondent-driven sampling methodology, which consisted of selecting adolescents as “seeds” to serve as the initial contacts for recruitment (see [26] for detailed description of sample recruitment methods). Written consent was obtained from adolescent participants aged 18 years and over in every site except Shanghai, where the age of majority was 16 years. For adolescents younger than 18 years (or 16 years in Shanghai), a combined written parental/guardian consent and child assent form was signed. A parent or guardian could include anyone who had legal authority over the child, which in some cases meant directors of homeless shelters or the Social Services Administration for foster children in Baltimore. All research protocols were approved by the Johns Hopkins Bloomberg School of Public Health IRB, and subsequently at each site’s human ethics review committee.

### Survey measures

#### Social factors

To examine the social context, we included seven measures of social factors across the contexts of family, peers, and neighborhoods. We included two measures of adult social support, each consisting of four items with a range from 0–12; one that assessed the extent of support from a male adult at the home (alpha range .75 to .84), and the other that assessed the extent of support from a female adult at the home (alpha range .87 to .97). Peer support consisted of a 6-item scale from 0 to 18 that assessed the extent of support from at least one friend (alpha range .69–.88).

We also included three different measures of individual perceptions and experiences of their neighborhoods. Sense of belonging was measured using a 3-item scale with a range from 0–9 to assess participants’ perceptions of their own level of connection to their neighborhood (alphas .78–. 85). Perceived fear was a 6-item scale from 0 to 18 that measured participants’ level of fear in their daily lives (alphas .77 to .87). Meanwhile, witnessing community violence consisted of nine items that were summed which asked respondents about how often they saw various violent acts in their neighborhood within the past 12 months (alphas .69 –. 87). Table 1 describes the items in each scale.

**Table 1:** Social factor measures

Social factor indicator	Component items
Adult social support	
Caring male adult at home	There is a male presently in your home who:
	- expects you to follow the rules.
	- believes that you will be successful.
	- you can turn to when you have a problem.
	- listens to you when you have something to say. *12*
Caring female at home	There is a male presently in your home who:
	- expects you to follow the rules.
	- believes that you will be successful.
	- you can turn to when you have a problem.
	- listens to you when you have something to say.
Caring adult at school	Has there been a teacher or some other adult in your school who
	- really cared about you.
	- told you when you do a good job.
	- noticed when you were not there.
	- always wanted you to do your best.
	- listened to you when you had something to say.
	- who believed that you would be successful.
	Responses: Never True (0) Sometimes True (1) Often True (2) Always True (3); Range: 0-12
Peer social support	
Social support	I have at least one friend:
	- that I can trust.
	- who would lend me money if I needed it.
	- I can talk to about family problems or real personal problems.
	- that I find it easy to talk to about sex.
	- I would turn to if I were in trouble.
	- who accepts me for who I really am.
	Response Options: Agree a lot (0) Agree a little (1) Disagree a little (2)
	Disagree a lot (3); Range: 0-18
Neighborhood social factors	
Sense of belonging	- I feel connected to most people in this neighborhood.
	- I know most of the people in this neighborhood.
	- I feel like I am part of a community in this neighborhood.
	Response Options: Agree a lot (0) Agree a little (1) Disagree a little (2) Disagree a lot (3); Range: 0-18
Perceived fear	How afraid are you:
	- of being attacked or robbed when you are out with other people in your neighborhood at night?
	- of being attacked or robbed when you are out alone in your neighborhood at night?
	- when you are on the street in your neighborhood during the day?
	- in your neighborhood when you are on your way to school or work?
	- when you are at school or work?
	- when you are at home in your house or apartment?
	Response Options: Not at all (0) A little (1) Somewhat (2) Very fearful (3); Range 0–18
Witnessing community violence	In the past 12 months, how often:
	- did you hear guns being shot in your neighborhood?
	- did you see somebody get arrested in your neighborhood?
	- did you see drug deals in your neighborhood?
	- did you see gangs in your neighborhood?
	- did you see someone being beaten up in your neighborhood?
	- was your house broken into?
	- did you see somebody in your neighborhood pull a gun, knife, or other weapon on another person?
	- did you see someone get killed in your neighborhood?
	Response Options: Never (0) – Once or more (1); Range 0–8

#### Health care seeking

We included three dichotomous outcome measures of health care seeking including contact, need, and lack of use. For both need and lack of use, participants were asked to specify the reasons for a visit or the lack of a visit. Contact with a health care provider was measured with a single item, *During the past 12 months, how often did you contact a doctor, nurse, or other health care provider?* The responses were dichotomized to *never* or *one or more times*. Need for a health care provider and health care use were both single item dichotomous measures, *During the past 12 months have you ever been sick or needed to see a health care provider?* and *During the past 12 months, has there been a time when you had a health care problem but did not get care for it*. Participants who responded “yes” to health care need were asked to identify the reason(s) for the need, which could include sickness, annual physical/sports check up, chronic disease issue, reproductive health, HIV testing or treatment, STI diagnosis or treatment, mental health, injury accident or violence, alcohol or substance use and/or other. Participants who responded ‘yes’ to lack of use were asked to identify the reasons, which included location, distance, fees/payment, service hours, waiting time, staff attitude, lack of confidentiality, health problems connected to illegal activity and/or other. Participants in New Delhi and Shanghai who responded no to health care need followed a different skip pattern than other sites and were not asked directly about lack of use.

#### Demographics

We also measured age, gender, current school enrolment, and historical family structure (raised by two parents, raised by one parent; raised by other). These measures are presented in Table 2.

**Table 2:** Demographic and social characteristics

	Baltimore W % (U %, *N*)	Delhi W % (U %, *N*)	Ibadan W % (U %, *N*)	J’burg W % (U %, *N*)	Shanghai W % (U %, *N*)
*N*	456	500	449	496	438
Sex					
Male	46.4 (57.7, 263)	52.8 (50.0, 250)	45.2 (49.0, 200)	56.7 (54.8, 272)	48.0 (50.7, 222)
Female	53.6 (42.3, 193)	47.2 (50.0,250)	54.8 (51.0, 229)	43.3 (45.2, 224)	52.0 (49.3, 216)
Age group					
15–16	43.0 (55.3,252)	47.5 (56.8, 284)	49.4 (59.5,267)	50.6 (24.8,123)	41.9 (29.0,127)
17–19	57.0 (44.7, 204)	52.5 (43.2, 216)	50.6 (40.5,182)	49.4 (75.2, 373)	58.1 (71.0, 311)
Mean Age (SE)	W 16.8 (0.2)	16.6 (0.1)	16.6 (0.1)	16.6 (0.2)	16.9 (0.2)
	U 16.3 (0.1)	16.4 (0.1)	16.4 (0.1)	17.3 (0.1)	17.3 (0.1)
School enrollment					
Enrolled	81.0 (85.3, 388)	82.7 (85.6, 428)	81.4 (81.3, 364)	84.3 (82.2, 406)	37.2 (28.5, 125)
Not Enrolled	19.0 (14.7, 67)	17.3 (14.4, 72)	18.6 (18.7, 84)	15.7 (17.8, 88)	62.8 (71.5, 313)
Graduated of Not Enrolled	66.6 (70, 47)	42.5 (41.7, 30)	68.0 (69, 58)	42.9 (36.4, 32)	30.1 (31.3, 98)
Raised by					
Two Parents	48.2 (45.3, 206)	94.7 (94.6, 473)	72.8 (71.6, 320)	54.7 (54.55,270)	83.1 (82.9, 363)
One Parent	27.7 (23.1,105)	1.31 (1.4, 7)	7.4 (7.4, 33)	9.7 (9.7, 48)	1.8 (1.8, 8)
Other Relative or Non-Relatives	24.2 (31.2, 142)	4.0 (4.0, 20)	19.8 (18.6, 83)	35.6 (35.6, 176)	15.1 (15.1, 66)

*Abbreviations*: *J’BURG* Johannesburg, *W%* Weighted Percent, *U%* Unweighted Percent

### Analysis

Site differences in adolescent health care seeking behaviors were first examined using one-way analysis of variance. We then examined the gender differences in health care seeking within each site using t-tests. Given differences in the age distribution across sites, a post-stratification age weight developed and employed in the cross-site descriptive presentations. The results from these analyses are in Tables 3 and 4. Multivariate analyses were then conducted with a logistic regression model using *lack of health care use when needed* as the dependent variable, and each social factor as an independent variable controlling for the age, gender, school enrollment, and family structure. All analyses were done with Stata using complex design procedures to accommodate the non-independence of observations (i.e., the potential for intercluster correlation within recruitment chain). Weights were generated via the RDSII estimator to account for the intercluster correlation and were used in the analyses presented in Table 5.

**Table 3:** Health Care Utilization among Adolescents in last 12 months across Sites by Gender

	Baltimore Female W % (U %, *N*) Male W % (U %, *N*)	New Delhi Female W % (U %, *N*) Male W % (U %, *N*)	Ibadan Female W % (U %, *N*) Male W % (U %, *N*)	Johannesburg Female W % (U %, *N*) Male W % (U %, *N*)	Shanghai Female W % (U %, *N*) Male W % (U %, *N*)
Female (*n*) Male (*n*)	188 251	250 250	222 213	224 272	215 220
Contact with a health care provider in the last 12 months ***	83.0 (78.2, 147)** 64.8 (65.9, 164)**	51.5 (51.0, 127)** 63.9 (62.7, 156)**	36.5 (37.8, 84) 43.9 (44.6, 95)	63.0 (64.7, 145) 61.7 (58.8, 160)	42.5 (45.1, 87) 33.5 (36.4, 80)
Been sick or needed a health care provider ***	53.7 (51.1, 96)* 37.0 (39.8, 100)*	46.5 (50.0, 125)* 61.0 (60.8, 152)*	35.9 (38.0, 84)* 37.9 (37.1, 79)*	59.0 (61.6, 138)* 40.9 (44.1, 120)*	36.2 (37.4, 80)** 22.6 (22.7, 50)**
Reasons for Seeking Care in the last 12 months (among those who report being sick or needing a health care provider)					
Female (*n*) Male (*n*)	97 96	124 147	89 82	138 119	79 49
Sickness (e.g. flu, fever, diarrhea, etc.)	60.4 (51.6, 50) 63.3 (43.8, 42)	65.6 (67.0, 83) 71.2 (68.0, 100)	48.5 (52.8, 47) 48.4 (51.2, 42)	74.9 (75.4, 104)*** 48.1 (53.9, 64)***	85.3 (91.1, 72) 78.2 (77.6, 38)
Annual physical/sports check-up	26.5 (25.8, 25)** 14.5 (21.9, 21)**	12.5 (12.1, 15) 14.4 (16.3, 24)	7.0 (6.7, 6) 13.2 (11.0, 9)	4.7 (3.6, 5) 5.8 (8.4, 10)	15.9 (13.9, 11) 11.9 (14.3, 7)
Chronic disease issue (diabetes, asthma, etc.)	10.0 (5.2, 5)*** 2.3 (5.2, 5)***	9.0 (8.9, 11) 4.0 (2.7, 4)	1.7 (2.3, 2) 1.4 (1.2, 1)	8.1 (9.4, 13) 7.4 (4.2, 5)	1.6 (2.5, 2) 5.6 (4.1, 2)
Reproductive health (contraceptives/condoms, pregnancy testing, etc.)	4.7 (6.2, 6)** 0.1 (1.0, 1)**	1.6 (0.8, 1) 2.0 (1.4, 2)	0 (0, 0) 4.6 (2.4, 2)	10.0 (6.5, 9)*** 1.0 (1.7, 2)***	1.0 (1.3, 1)*** 5.0 (2.0, 1)***
HIV testing or treatment	2.9 (2.1, 2) 0 (0, 0)	0 (0, 0) 2.0 (1.4, 2)	0 (0, 0) 1.2 (1.2, 1)	1.2 (2.9, 4)*** 8.2 (7.6, 9)***	0 (0, 0)* 5.0 (2.0, 1)*
STI diagnosis or treatment	4.6 (3.1, 3) 2.9 (1.0, 1)	2.7 (2.4, 3)** 0.2 (0.7, 1)**	3.9 (3.4, 3) 5.5 (3.7, 3)	1.7 (2.9, 4)*** 4.7 (4.2, 5)***	0 (0, 0) 0 (0, 0)
Mental health (counseling, diagnosis, treatment) ***	5.7 (7.2, 7) 3.2 (4.2, 4)	3.1 (3.2, 4) 2.8 (2.0, 3)	0 (0, 0) 1.9 (1.2, 1)	0.5 (1.5, 2) 0.5 (0.8, 1)	1.7 (2.5, 2)** 9.3 (6.1, 3)**
Injury, accident or violence ***	8.0 (10.3, 10) 8.1 (8.3, 8)	18.4 (21.0, 26) 12.9 (15.0, 22)	3.4 (5.6, 5) 6.8 (7.3, 6)	4.5 (4.4, 6)* 12.5 (10.1, 12)*	10.7 (6.3, 5) 14.1 (10.2, 5)
Alcohol or substance use ***	0.7 (1.0, 1) 0.7 (1.0, 1)	1.6 (1.6, 2) 1.3 (1.4, 2)	1.1 (1.1, 1)** 0.3 (1.2, 1)**	1.3 (1.5, 2)* 7.3 (7.6, 9)*	4.8 (5.1, 4)* 12.2 (4.1, 2)*
Other ***	9.4 (19.6, 19) 15.7 (22.9, 22)	16.9 (16.1, 20) 9.6 (9.5, 14)	12.2 (11.2, 10) 10.5 (13.4, 11)	6.4 (10.9, 15) 6.5 (6.7, 8)	16.1 (13.9, 11) 20.0 (24.5, 12)
None ***	5.2 (7.2, 7)*** 10.3 (16.7, 16)***	11.4 (10.5, 13) 9.8 (8.2, 12)	20.0 (18.0, 16) 13.5 (12.2, 10)	1.7 (2.9, 4)*** 8.6 (6.7, 8)***	0 (0, 0) 2.2 (4.1, 2)

*p < .05; **p < .01;***p < .001

**Table 4:** Barriers to Health Care Utilization among Adolescents in last 12 months across Sites by Gender

	Baltimore Female W % (U %, *N*) Male W % (U %, *N*)	New Delhi Female W % (U %, *N*) Male W % (U %, *N*)	Ibadan Female W % (U %, *N*) Male W % (U %, *N*)	Johannesburg Female W % (U %, *N*) Male W % (U %, *N*)	Shanghai Female W % (U %, *N*) Male W % (U %, *N*)
Female (*n*) Male (*n*)	188 248	125 148	219 214	224 271	79 50
Needed but did not seek health care in the last 12 months***	23.4 (23.4, 44) 23.4 (25, 62)	17.3 (18.4, 23) 22.2 (25.0, 37)	9.9 (11.0, 24)** 18.6 (22.0, 47)**	31.6 (33.5, 75) 37.8 (35.8, 97)	20.1 (22.8, 18) 23.7 (22, 11)
Reasons for Not Seeking Care in the last 12 months (among those who needed but did not seek health care)					
Female (*n*) Male (*n*)	43 62	23 37	29 47	74 97	18 11
Location	16.5 (11.6, 5)** 5.4 (11.3, 7)**	31.0 (34.8, 8) 47.9 (35.1, 13)	1.9 (3.5, 1) 9.4 (12.8, 6)	12.1 (10.8, 8) 12.2 (14.4, 14)	5.5 (11.1, 2)* 0 (0, 0)*
Distance	12.1 (11.6, 5) 17.1 (11.3, 7)	27.9 (21.7, 5) 16.2 (13.5, 5)	7.8 (6.9, 3) 0 (0, 0)	14.6 (9.5, 7) 10.8 (9.3, 9)	22.8 (33.3, 6) 0 (0, 0)
Fees/payment	11.5 (14.0, 6) 6.5 (8.1, 5)	14.1 (30.4, 7) 13.1 (16.2, 6)	14.6 (13.8, 4) 12.7 (14.9, 7)	10.2 (13.5, 10) 10.3 (13.4, 13)	37.7 (27.8, 5) 7.7 (18.2, 2)
Service hours	5.1 (2.3, 1) 5.3 (6.5, 4)	13.2 (13.0, 3) 4.0 (2.7, 1)	0 (0, 0) 11.3 (8.5, 4)	9.6 (8.1, 6)*** 4.2 (7.2, 7)***	9.0 (16.7, 3) 11.1 (18.2, 2)
Waiting time	22.0 (16.3, 7)* 12.6 (12.9, 8)*	25.2 (30.4, 7) 10.7 (8.1, 3)	10.5 (6.9, 2) 19.3 (12.8, 6)	16.3 (20.3, 15)* 11.4 (15.5, 15)*	40.9 (27.8, 5) 35.8 (36.4, 4)
Staff attitude	9.5 (4.7, 2) 0.9 (1.6, 1)	8.3 (8.7, 2) 0 (0, 0)	0 (0, 0) 5.1 (4.3, 2)	9.9 (13.5, 10) 5.9 (10.3, 10)	22.0 (5.6, 1) 27.3 (18.2, 2)
Lack of confidentiality	0.6 (2.3, 1)* 1.9 (1.6, 1)*	7.5 (4.4, 1) 1.0 (2.7, 1)	1.8 (3.5, 1) 1.2 (2.13, 1)	19.3 (13.5, 19)** 6.9 (10.3, 10)**	3.5 (5.6, 1) 13.1 (18.2, 2)
Health problems were connected to illegal activities	3.0 (2.3, 1)* 1.2 (3.2, 2)*	16.4 (21.7, 5)** 3.8 (5.4, 2)**	5.8 (10.3, 3) 18.1 (12.8, 6)	14.6 (5.4, 4)** 2.7 (2.1, 2)**	0 (0, 0) 18.1 (9.1, 1)
Other	33.4 (44.2, 19) 46.8 (45.2, 28)	26.4 (17.4, 4) 35.9 (37.8, 14)	17.8 (20.7, 6) 20.4 (27.7, 13)	9.9 (14.9, 11) 16.3 (22.7, 22)	66.6 (50.0, 9) 45.9 (36.4, 4)

*p < .05; **p < .01;***p < .001

**Table 5:** Social Factors Associated with Not Seeking Care When Needed in the past 12 months

	Baltimore	Ibadan	Johannesburg
	aOR (aCIs)	aOR (aCIs)	aOR (aCIs)
	*n* = 415	*n* = 363	*n* = 462
Social Factors			
Adult support from female	.87 (.77, .97)*	.98 (.91, 1.06)	.96 (.94, 1.00)
Adult support from male	.99 (.96, 1.03)	1.00 (.91, 1.09)	.95 (.91, 1.00)*
Peer social support	.92 (.84, 1.00)	1.02 (.89, 1.17)	.98 (.94, 1.01)
Perceived fear	1.08 (1.01, 1.15)*	1.04 (.97, 1.11)	1.07 (1.03, 1.11)**
Community Social belonging	1.04 (.88, 1.23)	.90 (.72, 1.12)	1.10 (1.01, 1.19)*
Witnessing Community Violence	1.06 (1.02, 1.09)**	1.07 (1.01, 1.13)*	1.08 (1.05, 1.12)***

*p < .05; **p < .01;***p < .001

*aOR* Adjusted Odds Ratio, *aCI* Adjusted Confidence Interva

## Results

### Demographics

Table 2 displays the demographic characteristics of the survey samples across all the sites. The analytic sample is comprised of 2339 respondents across the five sites (Baltimore *n* = 456; Delhi *n* = 500; Ibadan *n* = 449; Johannesburg *n* = 496; Shanghai *n* = 438). Across all sites the mean age of participants is 16.7 and 47.5 % of respondents are female. With the exception of Shanghai, where only 37.2 % of participants were enrolled in school at the time of the survey, a majority of participants in each site were currently enrolled in school (81.0 % in Baltimore – 84.3 % in Johannesburg). Participants in Delhi, Ibadan and Shanghai were generally raised in two parent households (94.7 %, 71.0 % and 80.1 % respectively). When they were not, other relatives or non-relatives raised participants in Ibadan and Shanghai (19.3 % and 16.5 %, respectively). In Baltimore and Johannesburg about half of the participants (47.8 % and 50.6 %) were raised in two parent families with 27.5 % of Baltimore participants and 10.6 % of Johannesburg participants being raised in single parent households and 38.7 % of Johannesburg participants and 24.0 % of Baltimore participants being raised by non-parental relatives or non-relatives.

### Health seeking and reasons for seeking care

As observed in Table 3, there were large variations in the prevalence of adolescents who saw a health care provider in the past 12 months across sites and gender. While adolescents in Baltimore were the most likely to see a health care provider in the past 12 months (*p*<. 01), there was a significant difference between the proportions of females vs. males seeking care (83 % of females vs. 65 % of males, *p* < 0.01). Interestingly, in New Delhi and Ibadan, the reverse was true, where males were more likely to seek care compared to their female counterparts (64 % of males in New Delhi vs. 52 % of females and in Ibadan, 44 % of males sought care vs. 37 % of females, *p* < 0.05). When adolescents were asked about needing to see a health care provider because of sickness, a slightly different pattern emerged (see Table 3). Here, adolescents in Baltimore, New Delhi and Johannesburg were the most likely to report needing to see a healthcare provider compared to adolescents from Ibadan and Shanghai (*p*<. 01). Approximately 61 % of males in New Delhi responded affirmatively, followed by females in Johannesburg (59 %), and females in Baltimore (54 %). Adolescents who were least likely to need a health care provider for sickness were male adolescents in Shanghai (22 %) and female adolescents in Ibadan (36 %).

Among those who reported seeing or needing a health care provider, a question was asked about their reasons for wanting to seek care. As observed in Table 3, the vast majority of adolescents across sites and gender indicated ‘sickness’ as their top reason, which could include having the flu, fever, or any number of illness symptoms. While there were little gender differences observed across most sites in the prevalence of adolescents who indicated ‘sickness’ as their main reason for seeking care, in Johannesburg, there was a significantly higher proportion of females (75 %) compared to males (48 %) who responded affirmatively to ‘sickness’, *p* < 0.001. Other reasons for seeking care varied greatly by both site and gender. In Baltimore, for example, more than a quarter of females (26.5 %) indicated seeking care because of an annual physical or sports check-up compared to only 15 % of males, *p* < 0.01; in New Delhi, a substantial proportion of both females (18 %) and males (13 %) indicated seeking care because of injuries, accidences or violence. Interestingly, while seeking care for reproductive health services was quite low across sites, in Johannesburg, 10 % of females indicated they needed care for such services compared to only 1 % of their male counterparts, *p* < 0.001.

### Barriers for seeking health care services

While adolescents in Baltimore, New Delhi, and Johannesburg are likely to seek care for a sickness, at the same time, a fairly large proportion of adolescents indicated that even when they needed health care, they didn’t seek it in the past 12 months. Shanghai, overall, had more young participants reporting that they did not seek care when needed (*p*<. 01). In Johannesburg, in particular, more than 30 % of both male and female adolescents did not seek care even when they knew it was needed. Similarly, nearly a quarter of both male and female adolescents in Baltimore and Shanghai indicated not seeking needed care. Interestingly, Ibadan was only site that had significant gender differences. While nearly 20 % of males indicated not seeking needed care in the past 12 months, less than 10 % of females reported similar responses (*p* < 0.01).

When asked about the reasons for why they didn’t seek care, there was little consistency except for the fact that most adolescents responded that ‘other’ reasons prevented them from seeking care. In Baltimore, 22 % of females indicated that the waiting times were a big barrier, whereas males indicated that distance was more of a factor (17 %). In New Delhi, both males and females indicated that location was a big barrier (31 % for females and 48 % among males); in Ibadan, however, fees/payment was indicated by nearly 15 % of females and 13 % of males, and nearly 20 % of males reported that not seeking care was related to their health problems being connected to illegal activities. In Johannesburg, one of the biggest barriers for females --- but not males – was lack of confidentiality (19 % for females; 7 % for males, *p* < 0.01). Finally, in Shanghai, with the exception of ‘other’ reasons, female adolescents were more likely to cite fees/payment (38 %) and waiting time (41 %) as barriers. Males in Shanghai also cited more waiting times (36 %) as well as staff attitudes (27 %) as barriers.

Given that such a high proportion of adolescents indicated ‘other’ as their main reason for not seeking care, we analyzed the qualitative data in the first phase to understand more about their perceptions about the factors that influence their health care seeking.

### Factors that influence health care seeking: findings from qualitative phase

#### Lack of trust

Across all five sites, the lack of trust that adolescents had not only for health care providers, but also for any adult in the community, was a key factor that prevented them from seeking care. With the exception of adolescents in New Delhi, adolescents all felt that health care providers either were not well equipped to help them or were rude and judgmental, which created an overall lack of trust in their services. In fact, one 18-year old female from Johannesburg who actually did seek services for an STI laughed about her experience:

*They just told me that I have an STI but never treated me and they referred me to the general hospital*

*I: What happened?*

*I just stayed home (Laughing)! They shouted at me (IDI, female adolescent, Johannesburg)*

Similarly, in Baltimore, some of the girls felt that health care providers tried to get them to use birth control just to reduce their population, and ignored them when it gave them problems.

*I actually think that birth control messes us up. I think they need to stop pushing birth control so hard because it’s messing up our insides. I think it’s on purpose, though. My cousin, she’s on birth control right now and she won’t stop bleeding. She keeps going to the doctors telling them what’s going on and they say it’s normal. I think they need to stop pushing it so hard. OK, you don’t want a high rate of population, but it’s messing up our insides. It really is. (IDI female adolescent, Baltimore)*

In Ibadan, one key informant reported that even when an adolescent sought services after she had been raped, the service provider blamed her for the incident.

*I remember one case – the girl that got raped, fortunately she got home and told her mother.... she was treated as if – oh you went out and did something and now you are lying that you got raped”. “. Her mother called me and I got involved and took her to UCH but the way she was handled, I got appalled to myself. (Key Informant, Ibadan)*

#### Embarrassment and stigma

Related to the lack of trust in health care providers, adolescents also often felt embarrassed and stigmatized asking for help from anyone for a health problem. In Baltimore, this was especially true among male adolescents who felt that they had to prove they were tough ‘on the streets’ and handle health challenges on their own.

*Unfortunately there are stigmas associated with that. To talk with a counselor or a psychologist or psychiatrist means that you are crazy. … And as such you are seen as vulnerable. Being seen as vulnerable on the street, especially if you’re someone who has to be on the streets, is not good because being seen as vulnerable could make the difference between life and death for you. (Key informant, Baltimore)*

Like the males in Baltimore, the discrimination perceived by migrant youth in Shanghai also seemed to influence males’ perception about their own reputation and the importance of not seeking health care services to preserve their sense of independence and strength.

*There are services provided, but for migrants, they are afraid of losing faces, so they don’t want to go into the neighborhood health committee {the local health center} for consulting. (Photovoice, male discussion, Shanghai)*

Notably, in Shanghai, many adolescents reported not wanting to go to the Neighborhood Health Committees (a local health facility that they knew provided contraceptives) as they would not only know many of the individuals who worked there, but because the local Shanghaies were perceived as being much more ‘traditional,’ they felt that they would be judged for seeking any contraceptive method.

Among females in Baltimore, embarrassment over being touched or observed by a health care provider was more frequently discussed. One female adolescent, in fact, didn’t know which was more embarrassing: to be touched by a male or a female health provider:

*I don’t like doctors touching me period. It’s uncomfortable. Because I don’t know whether I want a male doctor or a female doctor. You don’t want a female doctor because she might be gay.*

In both Ibadan and Johannesburg, adolescents and key informants mentioned the fear of being mocked by health care providers if they sought care for a problem that was caused by engaging in risky or unacceptable behaviors.

*Most youth feel that each time they go to a provider they will be judged or they would know the health care provider – and then they’d feel guilty that they have this health problem. Around the health care provider, they’d feel too young to have this health problem…. Actually, so many youth will not come out to tell a health care provider that they have been raped or anything as they believe they would only get trouble from the provider (Key informant, Ibadan)*

In Johannesburg, key informants felt that this was especially prevalent among HIV infected adolescents, who believed providers, were automatically going to assume they were behaviorally infected.

*And some think, that everyone who is HIV positive has it because they have been sleeping around -- but most of the children or these young people they are perinatal infected…(Key informant, Johannesburg)*

In Ibadan, key informants mentioned that confidentiality was especially important for adolescents and even if an adolescent had been sexually coerced and then contracted an STI, she would rather keep that information than face the potential risk of having a health care provider discuss her situation with her parents or any other adults in the community.

#### Lack of support from parents

In Baltimore, Johannesburg, and New Delhi, adolescents and key informants report that getting parents or guardians to participate or be available for health care visits was difficult as they were mostly unavailable or unwilling. Adolescents from Baltimore reported that because they need their parents to make appointments for them (for health care outside of reproductive health), they simply did not receive care.

*Because I’m only 15, I can’t make any of my own appointments. For my OB doctor, I can do that myself because I’m old enough, I guess…. But other doctors, no. (female adolescent, Baltimore)*

In Baltimore, fathers felt that they were particularly at fault since they admitted not wanting to seek formal health care themselves and were indirectly passing these feelings onto their children.

*One of the things about us men – is that we don’t like to go to the doctor. That’s bad because that cycle is passed down to our children, be it a boy or a girl. They may feel that they don’t want to go to the doctor, but I think it has something to do with that.*

In New Delhi, key informants mentioned that to receive services from any government health facility, a parental signature is required – which particularly affects girls wanting to seek help for reproductive health problems. In fact, several key informants described that even though girls know of where the facilities are located, and what services are offered, their fear of their parent’s reaction is often too strong to be overtaken for the sake of receiving needed health care services.

### Multivariate analysis of social and neighborhood factors on health care seeking

Table 5 displays the factors associated with not seeking health care when needed across Baltimore, Johannesburg, and Ibadan; New Delhi and Shanghai could not be included because of the differences in survey skip patterns in these sites. Interestingly, receiving adult support from home is influential to both adolescents in Baltimore and Johannesburg but not necessarily in the same manner. In Baltimore, adolescents who received adult support from a female at home was associated with a lesser likelihood of *not* seeking needed care (*p* < 0.05); for adolescents in Johannesburg, however, it was receiving adult support from a male at home that mattered (*p* < 0.05). At the neighborhood level, there were two factors that were particularly relevant to health seeking among adolescents: witnessing community violence and perceived fear. In all three sites, adolescents who witnessed community violence were more likely to report *not* seeking needed health care (*p* < 0.01). Perceived fear was also an important social factor, as young people in Baltimore and Johannesburg were more likely to *not* seek needed health care if they perceived higher levels of fear in their everyday lives (*p*<. 05). Interestingly, for adolescents living in Johannesburg, those who felt a greater sense of community violence were actually more likely to *not* seek needed health care (*p* < 0.05). Across all three sites, surprisingly, peer support was not associated to health seeking.

## Discussion

The primary aims of this study were to examine how adolescent health care seeking behaviors vary across sites, as well as how the influence of social factors on health care seeking behaviors vary among adolescents across urban sites. Our results show that health care seeking behaviors not only differed greatly across sites, but also between males and females. Females in Baltimore were actually the most likely to seek health care services, whereas males in Shanghai were the least likely. In general, research has shown that female adolescents are more likely to seek health care services compared to their male counterparts [16, 17, 27], and one study found that boys and men only seek care when the ‘need’ has led to significant personal consequences [28]. In our study, however, the reverse was true in both New Delhi and Ibadan, where more male adolescents sought health care services in the past 12 months compared to females. Qualitative data from both of these sites may shed some light as to why we observe this ‘exception.’ For instance, in both sites, female adolescents in New Delhi and Ibadan were quite restricted from leaving their homes in comparison to boys, who were perceived to have much greater freedom [29]. It seems very plausible, then, that in New Delhi and Ibadan, male adolescents are more likely to seek health care services compared to females because they simply have more freedom in mobility.

The study also showed that if adolescents are going to seek care, they are most likely going to seek care for a sickness, and this was true across sites and gender. This finding is consistent with what we see among adults, which suggests that seeking care is affected by the nature of the problem and how individuals perceive the problem [30]. With regards to the ‘nature’ of the problem, research shows that adults seek health care less often for intimate problems [31] and for problems perceived as ‘stigmatizing’ [32]. Given that the findings from this study showed that adolescents were least likely to report needing health care for reproductive and mental health problems, it is likely that the same health seeking behavior practices among adults apply for adolescents.

Even when adolescents need health services, it was interesting to find that more than 20 % of adolescents from Johannesburg, Baltimore, and Shanghai did not seek such needed services. To understand the reasons behind adolescent health care seeking behaviors, findings from both the qualitative and quantitative data provide at least some explanation. From the qualitative findings, trust was actually a big issue, as many adolescents felt that there was no adult – or provider – who could really help them. Indeed, previous research has also indicated that trust, even over the specific need for help, is a key determinant for whether a young person seeks help [27]. Studies conducted in the United States have also documented that Blacks have a much greater distrust of physicians and the health care system in comparison to Whites, largely due to personal experiences with racism, their knowledge of a history of racism in the healthy care system, and the social and cultural distance between Black patients and White physicians [33, 34]. Since issues around trust were especially prevalent among adolescents in Baltimore and Johannesburg – the two sites that have a long history in racial discrimination – it may also be that this lack of trust is deeply rooted and still propagated within the Black community. The qualitative data also showed that among adolescent males in particular, stigma was an important factor that affected their health care seeking behaviors. Somewhat similar results have been observed in Brazil, where authors found that adolescents with chronic health care conditions frequently viewed these conditions as a sign of personal weakness or failure, which in turn led to adolescents not wanting to seek needed health care services [35]. The fact that adult males also exhibited this same type of feeling – not liking to see the doctor – may perpetuate this notion that seeking health care services is ‘for the weak,’ although the adult males never described it in this way.

Turning to the findings from the multivariate analyses, it was particularly interesting to observe the predominance of fear and violence as determinants of health care seeking among adolescents. Indeed previous studies on adults have shown similar findings, suggesting that perceptions of fear, violence, and safety on a neighborhood level affect health-seeking patterns across age, gender, and setting. On a family level, our findings demonstrate that when adolescents feel supported by an adult in the home (female caregivers in Baltimore and male caregivers in Johannesburg), adolescents make better use of health care services. While the research on the influence of parental support and adolescent health care seeking behaviors is quite limited, it was somewhat surprising to find that the type of adult support that mattered to health seeking differed between these two sites. This may be reflective of both household structure and cultural parenting norms. In both sites, for example, a substantial proportion of adolescents are being raised either in single-parent homes or by other relatives/non-relatives. In Baltimore, we know that if adolescents are being raised in single-parent households, they are most likely living with their mothers [36]. In Johannesburg, while it might not necessarily be related to being raised by a single father, in most sub-Saharan African settings, fathers are the authoritative figures and the decision-makers of the household. According to Nsamenang [37], who has written substantially about the role of the father in Cameroon:

*The father assumes a crucial role in problem solving and protection of the family by exercising a moderating influence on family interactions with the external world. He is expected to be the first person to be consulted or informed of any trouble or major change in a child’s life. (p. 4).*

Indeed, more research is needed to better understand how parental/caregiver support, whether it be from the mother or father, influences adolescent health seeking.

The study has a number of important limitations. First, while respondent-driven sampling was a specific technique that was used to recruit adolescents from all diverse social backgrounds, each site’s sample is not representative of the general adolescent population in the particular cities. Some of the sites, such as New Delhi and Ibadan, may have also been constrained by the lack of exposure of respondents to computers and specifically ACASI. This may have affected the applicability of the technique to elicit accurate survey responses among the participants. Finally, it was unfortunate that participants in New Delhi and Shanghai followed a different skip pattern than what was followed in other sites, and as a consequence, we could not analyze their responses about their lack of health service use.

## Conclusions

Despite these limitations, this study confirms that adolescent health care seeking patterns are not universal and vary substantially by setting and gender. Complex social forces in both the neighborhood and family environments have an enormous influence on such behaviors. While previous efforts have focused on making health clinics ‘youth-friendly’ as a way to increase service utilization among adolescents, this study suggests that such initiatives will have little impact unless they are combined with strategies that target neighborhood safety and violence as well as building trust and support among adults in the family and the broader community. Further, while many youth-friendly initiatives focus on provider training as a way to help remove the barriers related to provider attitudes, parents are often ignored in such programs. This study, however, shows that parents have great influence on adolescent health care seeking both pragmatically (by requiring parents to make appointments) and through their support and communication with their adolescent children. Youth-friendly health initiatives, therefore, need to move beyond the walls of just the health care facility and address the factors within the family and community environments that in many ways exert more influence over adolescent health care seeking than even the facility itself. For there could be one of the best ‘youth-friendly’ clinics in a community, but if adolescents don’t feel safe in that community, and do not feel adults and parents are trustworthy or supportive, that clinic is likely to see very few, if any, adolescents from the community.

## Abbreviations

ACASI, audio computer assisted self interview; HIV, human immunodeficiency virus; IDI, in-depth interview; IRB, Institutional Review Board; RDS, respondent-driven sampling; STI, sexually transmitted infection; WAVE, wellbeing of adolescents in vulnerable environments

## Additional file

Additional file 1: Supplementary Selected Characteristics of Study Communities. (DOC 31 kb)
