# Supplementary material for: A mixed methods study to examine the influence of the neighborhood social context on adolescent health service utilization
Source: BMC Health Serv Res. 2016 Aug 24;16(1):433. doi: 10.1186/s12913-016-1597-x (PMC4997735; doi:10.1186/s12913-016-1597-x)
Supplement: Additional file 1: — Supplementary Selected Characteristics of Study Communities. (DOC 31 kb) [file 12913_2016_1597_MOESM1_ESM.doc]

**Supplementary** Selected Characteristics of Study Communities

| **City** | **Selected Community** | **Characteristics** |
| --- | --- | --- |
| Baltimore | East Baltimore | High prevalence of low income residents near the Johns Hopkins medical campus; majority of residents are African American |
| Shanghai | Sub-district | A suburban area located in the northwest of Shanghai, the size is 18.8 square kilometers with about 200,000 inhabitants and over half of inhabitants are migrants. |
| Johannesburg | Hillbrow | Densely populated inner city area (size is about 1 square kilometer with approximately 100,000 inhabitants); community is characterized by high levels of poverty and crime; made up of local Johannesburg residents and immigrants |
| Ibadan | Ibadan North Local Government Area, Oyo state | Third largest city in Nigeria; capital of Oyo state – within the city, a poor ‘inner city’ community was selected; predominant ethnic group was Yoruba |
| New Delhi | A slum in one of Delhi’s four districts (South Delhi) bordering the state of Haryana | Large slum community inhabited by migrant families from different parts of the country, overwhelmingly poor, lacking basic facilities, such as sanitation and water |
